# A mutated xylose reductase increases bioethanol production more than a glucose/xylose facilitator in simultaneous fermentation and co-fermentation of wheat straw

**DOI:** 10.1186/2191-0855-1-4

**Published:** 2011-03-28

**Authors:** Kim Olofsson, David Runquist, Bärbel Hahn-Hägerdal, Gunnar Lidén

**Affiliations:** 1 Department of Chemical Engineering, Lund University, P.O. Box 124, SE-221 00 Lund, Sweden; 2 Department of Applied Microbiology, Lund University, P.O. Box 124, SE-221 00 Lund, Sweden; 3 Fujirebio Diagnostics AB, Elof Lindälvs gata 13, PO Box 121 32, SE-402 42 Göteborg, Sweden

## Abstract

Genetically engineered *Saccharomyces cerevisiae *strains are able to ferment xylose present in lignocellulosic biomass. However, better xylose fermenting strains are required to reach complete xylose uptake in simultaneous saccharification and co-fermentation (SSCF) of lignocellulosic hydrolyzates. In the current study, haploid *Saccharomyces cerevisiae *strains expressing a heterologous xylose pathway including either the native xylose reductase (XR) from *P. stipiti*s, a mutated variant of XR (mXR) with altered co-factor preference, a glucose/xylose facilitator (Gxf1) from *Candida intermedia *or both mXR and Gxf1 were assessed in SSCF of acid-pretreated non-detoxified wheat straw. The xylose conversion in SSCF was doubled with the *S. cerevisiae *strain expressing mXR compared to the isogenic strain expressing the native XR, converting 76% and 38%, respectively. The xylitol yield was less than half using mXR in comparison with the native variant. As a result of this, the ethanol yield increased from 0.33 to 0.39 g g^-1 ^when the native XR was replaced by mXR. In contrast, the expression of Gxf1 only slightly increased the xylose uptake, and did not increase the ethanol production. The results suggest that ethanolic xylose fermentation under SSCF conditions is controlled primarily by the XR activity and to a much lesser extent by xylose transport.

## Introduction

The yeast *Saccharomyces cerevisiae *has been extensively engineered for ethanolic fermentation of the pentose sugar xylose either by introducing genes encoding xylose reductase (XR) and xylitol dehydrogenase (XDH), or by introducing the gene encoding xylose isomerase (XI) (Hahn-Hägerdal et al. 2007; Van Vleet and Jeffries 2009; Matsushika et al. 2009). The aim is to achieve economically feasible ethanolic fermentation of hardwood and/or agricultural lignocellulose feedstock, since these raw materials have a high content of pentose sugars, primarily xylose (up to 20% of the dry matter) (USDE-database). Still xylose fermentation with recombinant *S. cerevisiae *is significantly less efficient than hexose fermentation. Among others this has been ascribed to the difference in cofactor preference of XR and XDH, which results in xylose to xylitol conversion rather than ethanolic fermentation (Bruinenberg et al. 1983). Site directed mutagenesis has been applied on the XR to change the co-factor affinity, e.g. (Watanabe et al. (2007)). A different approach was used by (Runquist et al. 2010a) who arrived at a mutated version of the XR with changed kinetic properties using a random method in combination with a selection system. The mutant XR (N272D) from *Pichia stipitis *(mXR) has an increased ratio of NADH/NADPH utilization and an order of magnitude higher *V*_max _compared to the native enzyme. The introduction of mXR in *S. cerevisiae *otherwise engineered for xylose fermentation translated directly into increased ethanol yield and ethanol productivity and reduced xylitol formation in synthetic medium.

Slow xylose fermentation has also been ascribed to be the less efficient xylose transport. In *S. cerevisiae *xylose and glucose compete for the same transport systems (Kilian and Uden 1988; Meinander and Hahn-Hägerdal 1997) and the affinity for xylose is orders of magnitude lower than for glucose (Kötter and Ciriacy 1993; Saloheimo et al. 2007; Gárdonyi et al. 2003). Several homologous and heterologous xylose transporters have been expressed in *S. cerevisiae *(Hamacher et al. 2002; Saloheimo et al. 2007; Runquist et al. 2009; Katahira et al. 2008; Hector et al. 2008). Among the heterologous transporters the glucose/xylose facilitator Gxf1 from *Candida intermedia *(Leandro et al. 2006) proved to have the highest transport capacity, which was reflected in the highest aerobic xylose growth rate (Runquist et al. 2010b). Gxf1 has also been expressed in the industrial xylose fermenting *S. cerevisiae *strain TMB3400 (Fonseca et al. submitted). Its presence increased xylose consumption in simultaneous saccharification and co-fermentation (SSCF) of acid-pretreated wheat straw, however, without increasing the ethanol yield.

Simultaneous saccharification and fermentation (SSF) (Takagi et al. 1977) has been established as a promising option for ethanol production from lignocellulosic materials (Olofsson et al. 2008a) since the overall ethanol yield has been reported to be higher than if the enzymatic hydrolysis and fermentation are carried out separately (SHF) (Wingren et al. 2003). Furthermore it also been established that xylose consumption increases in SSF (Öhgren et al. 2006; Olofsson et al. 2008b), which has therefore been re-named SSCF also to include co-fermentation of hexose and pentose sugar.

The current study was undertaken to investigate to what extent the presence of mXR instead of XR would allow ethanolic fermentation of the additional xylose taken up by strains carrying the Gxf1 facilitator (Fonseca et al. submitted). Therefore, isogenic haploid *S. cerevisiae *CEN.PK strains expressing a heterologous XR/XDH/XK pathway were constructed. In addition to the control strain carrying the native XR, strains carrying mXR, Gxf1 and both mXR and Gxf1 were generated. The strains were assessed in SSCF of acid-pretreated wheat straw. The presence of mXR significantly increased xylose uptake and ethanolic xylose fermentation and reduced xylitol formation. In contrast, Gxf1 either alone or together with mXR, at most increased xylose uptake with about 10% leaving the ethanol formation unchanged.

## Materials and methods

### Raw material and pretreatment

Wheat straw, locally harvested and dried in the field (Johan Håkansson Lantbruksprodukter, Lunnarp, Sweden), was milled and sieved into 1- to 10-mm pieces and soaked overnight in 0.2% (v/v) H_2_SO_4 _at room temperature in closed barrels at a solids loading of 10% (wt/wt). The impregnated straw was pressed to 300 bars and reached a dry matter content of 50%. It was subsequently steam-pretreated batchwise at 190°C for 10 min in a 10-L reactor described by (Palmqvist et al. (1996)). The pretreated material was stored at 4°C. The composition of the pretreatment slurry is presented in Table 1. The water-insoluble solids (WIS) and liquid fractions were analyzed using National Renewable Energy Laboratories (NREL) standard procedures (Sluiter et al. 2008a, b). The WIS content of the pretreated slurry was determined to be 13% (wt/wt) by washing the fibers with deionized water over filter paper.

**Table 1:** Composition of the pretreated wheat straw material (WIS-content: 13.1%).

**Content in solid fraction (% WIS)**		**Content in liquid fraction (g L^-1^)**	
	
Glucan	53.3	Glucose^a^	9.3
Xylan	3.3	Xylose^a^	35.7
Lignin	34.5	Furfural	2.2
		HMF	< 0.1
		Acetic acid	4.3

^a^Both monomeric and oligomeric forms are included.

### Strain construction

Standard molecular biology techniques were used for all cloning procedures (Sambrook et al. 1989). Fermentas GeneJet plasmid miniprep kit (Fermentas, Vilnius, Lithuania) was used for plasmid extraction and Qiagen Gel Extraction Kit (Qiagen GMBH, Hilden, Germany) was used to extract DNA from agarose gels. Restriction enzymes were obtained from Fermentas. The lithium acetate method was used for transformation of *S. cerevisiae *(Gietz et al. 1995). Homologs of the previously described xylose-utilizing strain TMB3422 and TMB3424 (Table 2) were constructed expressing the Gxf1 glucose/xylose transporter. Plasmid YIpDR7 and YIpOB8 were linearized using *EcoRV *and transformed into strain TMB 3043-Gxf1, yielding strains TMB3425 and TMB3426, respectively (Table 2).

**Table 2:** *S. cerevisiae *strains and plasmids used in this study.

Strains and Plasmids	Relevant Genotype	Reference
Plasmids		
		
YIpOB8	*URA3 TDH3p-XYL1-ADH1t, PGK1p-XYL2-PGK1t*	(Bengtsson et al. 2009)
YIplac128	*LEU2*	(Gietz and Sugino 1988)
YIpDR1	YIplac128 *TDH3p*-*GXF1*-*CYC1t*	(Runquist et al. 2009)
YIpDR7	pOB8 XR N272D	(Runquist et al. 2010a)
		

*S. cerevisiae *strains		
		
TMB 3043	CEN.PK 2-1C Δgre3, *his3*::*PGK1p-XKS1-PGK1t, TAL1::PGK1p-TAL1-PGK1t, TKL1::PGK1p-TKL1-PGK1t, RKI1::PGK1p-RKI1-PGK1t, RPE1::PGK1p-RPE1-PGK1t*, *leu2*, *ura3*	(Karhumaa et al. 2005)
TMB 3043-Gxf1	TMB 3043, *leu2*::YIpDR1, *ura3*	(Runquist et al. 2009)
TMB 3422	TMB 3043, *leu2*::YIplac128, *ura3*::YIpDR7	(Runquist et al. 2010a)
TMB 3424	TMB 3043, *leu2*::YIplac128, *ura3*:: YIpOB8	(Runquist et al. 2010a)
TMB 3425	TMB 3043, *leu2*::YIpDR1, *ura3*::YIpDR7	This work
TMB 3426	TMB 3043, *leu2*::YIpDR1, *ura3*::YIpOB8	This work

### Cell propagation for SSCF

The recombinant xylose-fermenting strains *S. cerevisiae *TMB3422, TMB3424, TMB3425 and TMB3426 (Table 2) to be used in the SSCF were propagated in sequential cultures starting with a preculture in shake flask, followed by aerobic batch cultivation on glucose and finally aerobic fed-batch cultivation in wheat straw pretreatment liquid to improve inhibitor tolerance (Alkasrawi et al. 2006).

The yeast was inoculated in 300-ml flasks containing 100 ml media supplemented with 16.5 g L^-1 ^glucose, 7.5 g L^-1 ^(NH_4_)_2_SO_4_, 3.5 g L^-1 ^KH_2_PO_4_, 0.74 g L^-1 ^MgSO_2_·7H_2_O, trace metals and vitamins. The cells were grown for 24 h at 30°C and pH 5 in a rotary shaker at 180 rpm. Subsequently, aerobic batch cultivation was performed in a 2.5-L bioreactor (Biostat; A. B. Braun Biotech International, Melsungen, Germany) at 30°C. The working volume was 0.7 L, and the medium contained 20.0 g L^-1 ^glucose, 20.0 g L^-1 ^(NH_4_)_2_SO_4_, 10.0 g L^-1 ^KH_2_PO_4_, 2.0 g L^-1 ^MgSO_4_, 27.0 mL L^-1 ^trace metal solution and 2.7 mL L^-1 ^vitamin solution (Taherzadeh et al. 1996). The cultivation was initiated by adding 20.0 mL of the preculture to the bioreactor. The pH was maintained at 5.0 throughout the cultivation by automatic addition of 3 M NaOH. Aeration was maintained at 1.2 L min^-1^, and the stirrer speed was kept at 800 rpm. When the ethanol produced in the batch phase was depleted, the feeding of wheat straw pretreatment liquid was initiated. A total of 1.0 L of wheat straw pretreatment liquid was added starting with an initial feed rate of 0.04 L h^-1^, which was increased linearly to 0.10 L h^-1 ^during 16 h of cultivation. The aeration during the fed-batch phase was maintained at 1.5 L min^-1^, and the stirrer speed was kept at 800 rpm.

Cells were harvested by centrifugation in 700-mL flasks using a HERMLE Z 513K centrifuge (HERMLE Labortechnik, Wehingen, Germany) and resuspended in 9 g L^-1 ^NaCl solution to obtain a cell suspension for SSCF with 80 g dry wt L^-1^. The time between cell harvest and initiation of the SSCF was no longer than 3 h.

### SSCF

All SSCF experiments were carried out batch-wise in duplicates under anaerobic conditions using 2.5-L bioreactors (Biostat; A. B. Braun Biotech International, Melsungen, Germany; Biostat A plus; Sartorius, Melsungen, Germany) sterilized by autoclavation. The experiments were carried out with a WIS content of 7% with a total working weight of 1.4 kg. To obtain the initially desired WIS content in the bioreactor, the pretreated, undetoxified slurry was diluted with sterile deionized water. Before adding the pretreated slurry to the reactor, pH was adjusted to 4.8 with the addition of 10 M NaOH. All SSCF experiments were carried out at 32°C for 96 h. pH was maintained at 5.0 throughout the SSCF by automatic addition of 3 M NaOH, and the stirring rate was kept at 500 rpm. The SSCF medium was supplemented with 0.5 g L^-1 ^NH_4_H_2_PO_4_, 0.025 g L^-1 ^MgSO_4_·7H_2_O and 1.0 g L^-1 ^yeast extract. An initial yeast concentration of 4 g dry wt L^-1 ^was used. The enzyme preparation was Cellic CTec (Novozymes A/S, Bagsvaerd, Denmark) with a FPU (filter paper units) activity of 95 FPU g^-1 ^and a β-glucosidase activity of 590 IU g^-1^. The total amount of enzyme added to each SSCF experiment corresponded to 10 FPU (g WIS)^-1 ^and 62.1 IU (g WIS)^-1 ^β-glucosidase activity. Samples for high performance liquid chromatography (HPLC) analysis were taken repeatedly throughout the SSCF. All SSCF experiments were carried out in duplicates.

### Analysis and calculation

The dry weight (DW) of the 9 g L^-1 ^NaCl cell suspension (described above) was determined in duplicates from 10 mL samples centrifuged (1000 × g) for 5 min at 3000 rpm (Z200 A, HERMLE Labortechnik, Wehingen, Germany). Supernatants were discarded, and pellets were washed with 9 g L^-1 ^NaCl solution and centrifuged a second time. Pellets were dried at 105°C overnight and weighed. FPU activity (Adney and Baker 1996) and β-glucosidase activity (1 IU corresponding to conversion of 1 μM substrate min^-1^) (Berghem and Pettersson 1973) were determined as previously described and modified (Olofsson et al. 2010). Substrates and products from the SSCF experiments were quantified by HPLC (Olofsson et al. 2010).

The ethanol yield, *Y*_E/S_, was calculated on the basis of the total amount of fermentable sugars added to the SSCF, i.e., the sum of glucose and xylose present in the pretreatment slurry, including monomers, oligomers and polymers (glucan and xylan fibers). The theoretical mass of glucose released during hydrolysis is 1.11 times the mass of glucan (due to the addition of water). For xylose the corresponding number is 1.13 times the mass of xylan.

## Results

The current study aimed to evaluate the relative contribution of a mutated xylose reductase (mXR) (Runquist et al. 2010a) and a glucose/xylose facilitator (Gxf1) (Runquist et al. 2009) (Fonseca et al. submitted) to the fermentation of xylose in a simultaneous saccharification and co-fermentation (SSCF) set-up (Olofsson et al. 2008a) of pretreated wheat straw. Independently mXR (Runquist et al. 2010a) and Gxf1 (Runquist et al. 2009) have been shown to increase the ethanolic fermentation of xylose in synthetic medium. To allow the comparison of these two genetic traits in an isogenic strain background - in SSCF of pretreated non-detoxified wheat straw - four differently engineered xylose-utilizing CEN.PK strains were constructed and compared; the control strain TMB3424 (Runquist et al. 2010a) harboring the native XR, strain TMB3422 harboring mXR and generated by introducing YIpDR7 (Runquist et al. 2010a) in strain TMB3043 (Karhumaa et al. 2005), strain TMB3426 harboring Gxf1 and generated by introducing YIpDR1 (Runquist et al. 2009) in TMB 3043, and strain TMB3425 harboring both mXR and Gxf1 and generated by introducing both YIpDR7 and YIpDR1 in strain TMB3043 (Table 2).

**Figure 1:**
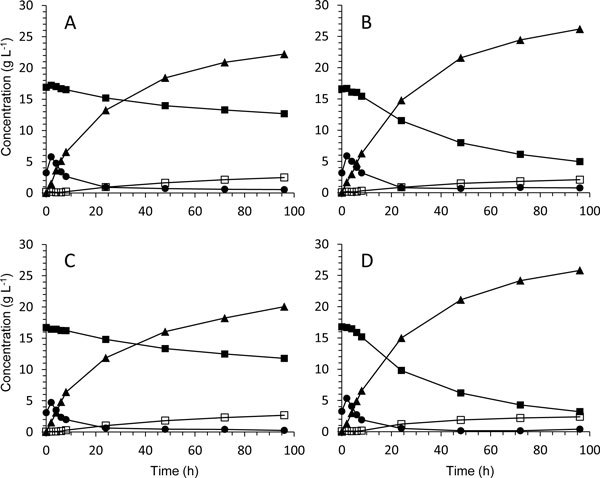
**Measured concentrations during duplicate batch SSCF of wheat straw with 7% WIS showing glucose (●), xylose (■), xylitol (□) and ethanol (▲)**. A: TMB3424 (native XR). B: TMB3422 (mutated XR). C: TMB3426 (native XR + Gxf1). D: TMB3425 (mutated XR + Gxf1).

The control strain displayed a relatively slow fermentation of xylose (Figure 1A) and had at the end of the SSCF only consumed 38% of the available xylose (Table 3). Furthermore about one third, 32%, of the consumed xylose was secreted as xylitol so that only about 25% of the available xylose was fermented and contributed to the final ethanol concentration, 22.2 g L^-1^.

**Table 3:** Summary of SSCF of wheat straw with 7% WIS after 96 h showing concentrations and yields (mean values of duplicate experiments). The same conditions (temperature, pH, yeast- and enzyme loading) were used in all experiments.

XR and Gxf1 expression (Strain)	Xylose (g L^-1^)	Xylitol (g L^-1^)	Glycerol (g L^-1^)	Ethanol (g L^-1^)	Xylose consumption^a^ (%)	Xylitol yield^b^ (%)	Ethanol yield^c^ (g g^-1^)
Native XR (TMB3424)	12.7 ± 0.9	2.5 ± 0.3	4.4 ± 0.1	22.2 ± 0.1	38	32	0.33
Mutated XR (TMB3422)	5.0 ± 0.6	2.1 ± 0.1	3.9 ± 0.3	26.2 ± 0.4	76	13	0.39
Native XR+Gxf1 (TMB3426)	11.8 ± 2.0	2.7 ± 0.8	4.1 ± 0.2	20.1 ± 0.1	42	31	0.30
Mutated XR+Gxf1 (TMB3425)	3.2 ± 0.5	2.4 ± 0.1	4.2 ± 0.1	25.9 ± 0.9	84	13	0.39

a. Based on to total amount of xylose (present both in the fibers and the liquid fraction).

b. Based on consumed xylose.

c. Based on total amount of available sugars (present both in the fibers and the liquid fraction).

When the native XR was replaced by mXR the xylose consumption was almost doubled from 38% to 76% (Table 3; Figure 1A and 1B). Additionally, the xylitol yield was reduced from 32% to 13%, which resulted in a 20% increased ethanol yield of 0.39 and a final ethanol concentration of 26.2 g L^-1 ^(Table 3). In the isogenic strain background the significantly improved ethanolic xylose fermentation directly reflects the difference between the kinetic properties of the native and the mutated XR (Runquist et al. 2010a). Both *V*_max _and the NADH/NADPH utilization ratio for mXR are an order of magnitude higher than for the native XR, which in SSCF translate to faster xylose utilization and significantly less xylitol secretion.

In contrast to the influence of mXR on xylose consumption and ethanol production the introduction of the glucose/xylose facilitator Gxf1 only marginally influenced SSCF of pretreated wheat straw (Figure 1A and 1C; Table 3). The small increase in xylose consumption observed in comparison to the control strain was not statistically significant, and the same applies for the changes in ethanol and xylitol yields.

The rather limited influence of Gxf1 when the currently used strain background was assessed in ethanolic xylose fermentation in SSCF was further demonstrated when mXR and Gxf1 were both introduced in the same strain. The mXR/Gxf1 strain displayed a substrate-consumption/product-formation pattern very similar to the mXR strain (Figure 1B and 1D). Again a slight increase in xylose consumption was observed, from 76 to 84% (Table 3). However, the final ethanol concentration, as well as the ethanol and xylitol yield, was the same as for the mXR strain.

## Discussion

The glucose/xylose facilitator Gxf1 from *C. intermedia *(Leandro et al. 2008) has been shown to increase xylose uptake and aerobic growth at low sugar concentrations in an laboratory xylose-utilizing CEN.PK strain (Runquist et al. 2009) as well as in the industrial xylose-utilizing TMB3400 strain (Fonseca et al. submitted). Similarly, the presence of the mutated (N272D) xylose reductase (mXR) from *P. stipitis*, increased xylose uptake and anaerobic growth (Runquist et al. 2010a) in synthetic medium. In addition, mXR shifted product formation from xylitol to ethanol. The current comparison using isogenic *S. cerevisiae *CEN.PK strains was undertaken to elucidate the relative contribution of these two beneficial genetic modifications on xylose consumption and ethanol and clarify if these genetic traits could act synergistically. Simultaneous saccharification and co-fermentation (SSCF) (Olofsson et al. 2008a) of non-detoxified pretreated wheat straw was chosen as experimental model, since it is an industrial medium, interesting for commercial ethanol production scale. Our investigation showed that in the CEN.PK strain background and in the SSCF set-up, mXR had a far greater influence on xylose consumption and product formation than Gxf1. The presence of mXR doubled the xylose uptake, decreased the xylitol yield by half and as a result increased the obtained ethanol yield in SSCF by about 20%. In contrast, Gxf1 at most increased the xylose uptake by 10% irrespective of the presence of XR and mXR, receptively.

SSF (simultaneous saccharification and fermentation), the forerunner of SSCF was originally designed as a means to generate low glucose concentration in the reactor to overcome glucose inhibition of cellulose hydrolysis (Takagi et al. 1977). It was later observed that this set-up also favored co-utilization of xylose when recombinant xylose-utilizing strains of *S. cerevisiae *were used (Olofsson et al. 2008b; Öhgren et al. 2006). In SSCF, the fermenting yeast is exposed to a high xylose/glucose ratio since the hemicellulose fraction is primarily hydrolyzed in the acid-pretreatment step (Olofsson et al. 2008a) while glucose is continuously released throughout the enzymatic hydrolysis. Enhanced co-utilization of xylose and glucose in SSCF is in accordance with numerous independent observations, which demonstrated that glucose in fact enhances xylose utilization at low but non-zero concentrations (Meinander et al. 1999; Pitkänen et al. 2003; Krahulec et al. 2010). This has been attributed both to activation of the enzymes of the lower glycolytic pathway (Boles et al. 1996), and to improved co-factor regeneration (Pitkänen et al. 2003). In addition the low glucose concentration in SSCF favors induction of high affinity hexose transporters, which also display high affinity for xylose (Pitkänen et al. 2003; Bertilsson et al. 2008). Therefore the fact that xylose uptake only increased by 10% in the Gxf1 strains may not only reflect the properties of the transporter, but may also result from the SSCF conditions.

When the Gxf1 transporter was expressed in the industrial *S. cerevisiae *strain TMB3400 and assessed in SSCF of acid-pretreated wheat straw similar to the current experimental set-up, the xylose uptake also increased by about 10% (Fonseca et al. submitted). The additional xylose taken up was stoichiometrically converted to xylitol and glycerol. Metabolic flux analysis (MFA) suggested that the presence of Gxf1 shifted the control of xylose catabolism from transport to down-stream catabolic reactions. The mXR mutant has a higher *V*_max _and higher NADH/NAPH selectivity ratio, which was shown to directly relate to increased anaerobic xylose growth and increased ethanol formation (Bengtsson et al. 2009; Runquist et al. 2010a). The current study was set up to investigate whether the presence of mXR would shift the control of xylose catabolism to transport. However, the results show that xylose catabolism downstream of transport still dictated the metabolic flux, and that an even faster xylose catabolism would be required to fully benefit from the increased xylose transport capacity. The presence of only Gxf1 resulted in slightly higher xylose consumption, which was not converted to ethanol. Instead somewhat less ethanol was produced, which was not seen when mXR was also expressed. This may reflect that transport exercises a slightly higher control in the strain harboring mXR because mXR has significantly higher activity than XR (Runquist et al. 2010a) which is in accordance with previous reports showing that transport becomes more controlling at higher XR activity (Gárdonyi et al. 2003).

During pretreatment and hydrolysis a spectrum of compounds that inhibit the cellular metabolism are released and formed and many of these compounds inhibit ethanolic fermentation (Almeida et al. 2007). *S. cerevisiae *strains with an industrial background are generally more inhibitor tolerant than haploid laboratory strains (Almeida et al. 2007). The haploid CEN.PK strain background was chosen in the current study to generate isogenic strains that permitted the assessment of the relative contribution of mXR and Gxf1, respectively, to ethanolic xylose fermentation in SSCF of pretreated wheat straw. The control strain carrying the native XR consumed 38% of the available xylose, whereas the mXR strain converted twice as much in the non-detoxified wheat straw. The conversion obtained with the mXR strain in fact compared well to that reported for the industrial XR/XDH based xylose fermenting strain TMB3400 in SSCF of pretreated wheat straw of a similar composition (Olofsson et al. 2008b).

Among the inhibitory compounds formed during pretreatment and hydrolysis, there are several which act as electron acceptors (Almeida et al. 2007). Such compounds have been shown to function as "redox sinks" able to alleviate the redox imbalance caused by the difference in cofactor preference of XR and XDH (Wahlbom and Hahn-Hägerdal 2002). This has been shown to reduce the xylitol yield in non-detoxified hydrolyzate with as much as three times in model SSF experiments compared to defined media (Olofsson et al. 2008b). For the mXR strain xylitol formation was reduced about 50% from 0.24 to 0.13 g g^-1 ^when compared with xylose fermentation in defined medium (Runquist et al. 2010a).

In conclusion, the current work investigated targeted metabolic changes for improved xylose fermentation in SSCF of undetoxified pretreated wheat straw. These kinds of investigations are important since strain-improvements are often considerably less pronounced in lignocellulosic hydrolyzates under process-like conditions. Due to the mutated XR the xylose uptake could be doubled along with a significant reduced xylitol yield, resulting in a substantial increase in the ethanol yield. It will be important to increase the final ethanol concentration further by increasing the WIS-content with a maintained ethanol yield for the economic viability of the process (Galbe et al. 2007). This is likely to require a combination of further strain development and improved process technology.

## Competing interests

BHH is co-founder and chairman of the board of C5 Ligno Technologies in Lund AB.

## Authors' contributions

KO participated in the design of the study, performed the experimental work and wrote the manuscript. DR participated in the design of the study, constructed the strains and commented on the manuscript. GL and BHH participated in the design of the study and commented on the manuscript. All authors contributed to the scientific discussion throughout the work and have read and approved the final manuscript.
